# Physicochemical Properties and Liposomal Formulations of Hydrolysate Fractions of Four Sea Cucumbers (Holothuroidea: Echinodermata) from the Northwestern Algerian Coast

**DOI:** 10.3390/molecules25132972

**Published:** 2020-06-28

**Authors:** Asmaa Mecheta, Amine Hanachi, Carole Jeandel, Elmira Arab-Tehrany, Arnaud Bianchi, Emilie Velot, Karim Mezali, Michel Linder

**Affiliations:** 1 Laboratory of Protection and Development of Coastal Marine Resources and Molecular Systematics, Department of Marine Sciences and Aquaculture, Faculty of Natural and Life Sciences, Abdelhamid Ibn Badis University Mostaganem, BP 227, National road N° 11, Kharrouba 27000, Mostaganem, Algeria; 2 Laboratoire d’Ingénierie des Biomolécules (LIBio), Lorraine University, 2, Forêt de Haye avenue TSA 40602, 54518 Vandœuvre CEDEX, France; amine.hanachi@univ-lorraine.fr (A.H.); carole.jeandel@univ-lorraine.fr (C.J.); elmira.arab-tehrany@univ-lorraine.fr (E.A.-T.); 3 UMR 7365 CNRS- Molecular Engineering and Articular Physiopathology, 9 Forêt de Haye Avenue, BP 20199, 54505 Vandœuvre-Lès-Nancy, France; arnaud.bianchi@univ-lorraine.fr; 4 Faculty of Pharmacy, Laboratory of Practical Work in Physiology, Lorraine University, Brabois-Health Campus, 7 Forêt de Haye Avenue, BP 90170, F-54505 Vandœuvre-lès-Nancy CEDEX, France; emilie.velot@univ-lorraine.fr

**Keywords:** sea cucumbers, PUFA, peptide, ACE, nanoliposome, phospholipids, Algerian coast

## Abstract

To promote the nutritional and pharmacological values of four sea cucumber species (*Holothuria poli, H. tubulosa, H. arguinensis*, and *H. sanctori*), harvested from the Algerian coast, we aimed to study their proximate composition, fatty acid profile and angiotensin-converting enzyme (ACE) inhibitory activity. Their phospholipids were also used to elaborate nanoliposomes and to encapsulate peptides obtained from the same source. After the physico-chemical characterization of nanoliposomes and peptides, in vitro analyses were realized. The four holothurian species showed a high amount of protein (49.26–69.34%), and an impressive lipid profile of 27 fatty acids, mainly composed of polar fatty acids (91.16–93.85%), with a high polyunsaturated fatty acids (PUFA) content (50.90–71.80%), particularly eicosapentaenoic acid (EPA) (5.07–8.76%) and docosahexaenoic acid (DHA) (4.86–7.25%). A high phospholipids amount was also found (55.20–69.85%), mainly composed of phosphatidylcholine (PC) (51.48–58.56%). Their peptide fractions exhibited a high ACE inhibitory activity (IC_50_ 0.30 to 0.51 mg/mL). The results also showed that the nanoliposomes do not induce cytotoxicity and cell death in human MSCs and no perturbation of proliferation for all the times and the tested concentrations, as well as the combined nanoliposomes and hydrolysates (HTS) at a concentration of 0.1 mg/mL. All four sea cucumbers show potential as a new source for omega-3, omega-6, and bioactive peptides.

## 1. Introduction

Recently, marine organisms-based products have attracted special attention due to their pharmacological potential related to their secondary metabolites [1,2,3]. Among marine benthic invertebrates, Holothurians (commonly known as sea cucumbers) have received particular attention due to the presence of biomolecules that have health benefits such as: (1) chondroitin sulfates [4]; (2) fucan sulfates [5]; (3) polysaccharides [6]; (4) triterpene glycosides (saponins) [7]. These four biomolecule types have applications in the pharmaceutical industry due to their broad panel of bioactivities, such as antibacterial, antifungal, antiprotozoal, anti-inflammatory, anticoagulant, antitumor, antioxidant, and antiviral activities [8,9,10]; (5) long-chain polyunsaturated fatty acids (PUFAs) that play an essential role in metabolic activities [11], coronary heart diseases, arthritis, wound healing, and blood clotting [12,13,14], and (6) peptides [15] which exhibit angiotensin-converting enzyme (ACE) inhibition [16,17,18]. Peptides’ biological activities decrease sometimes due to digestion or poor absorption [19,20]. One of the approaches applied to improve the intestinal absorption of peptides is encapsulation in nano- and submicron-sized vesicles [21], which can enhance the bioavailability of peptide-based drugs by virtue of their small size and high surface area [22,23]. Liposomes can be easily produced from food grade materials [24], and if the proper formulation and preparation methods are chosen, they can be biodegradable and non-toxic [25,26]. In a single vesicle, liposomes can carry both hydrophilic and hydrophobic components [24]. This allows them to encapsulate both polar and non-polar amino acids from low molecular weight protein hydrolysates [23]. Liposomes protect proteins from stomach acids and gastric protease, pepsin, and enzymes such as trypsin and chymotrypsin in the small intestine [23].

Sea cucumbers are soft-bodied marine invertebrates belonging to the phylum Echinodermata. Worldwide, more than 1716 species have been described, including 70 species currently used commercially and in aquaculture [10,27]. Most of the exploited species are deposit-feeders, collecting and assimilating a large quantity of organic detritus and soft sediments from the seabed. They contribute to the recycling and re-mineralization of organic matter, creating a significant “turnover” and thus play an important role in the oxygenation of the soft substrate [28,29,30].

Sea cucumbers are mostly harvested in China and other southeast Asian countries [31,32]. They are fishery products with high nutritional value. The commercially dried product known as “bêche-de-mer” is consumed as a functional and tonic food or for medicinal use, especially in traditional medicine in many Asian countries [33,34]. These organisms continue to be extensively exploited, and their industry has been booming since the 1980s due to a growing interest in their nutritional, biological and pharmaceutical properties. [9,34,35]. As a consequence, sea cucumber fisheries around the world are completely over-exploited [36], and Asian markets are now targeting new species, principally from the Mediterranean Sea and the NE Atlantic Ocean [37,38]. The main holothuroids found in Mediterranean shallow water areas belonging to the order Holothuriida [*Holothuria tubulosa*, *Holothuria poli*, *Holothuria forskali*, *Holothuria sanctori* and *Holothuria arguinensis*, an invasive species originating from the Atlantic Ocean [39], and to the order Synallactida (*Parastichopus regalis*) [40]. Holothuriida species are the main representatives of the benthic compartment of the *Posidonia oceanica* ecosystem in the Mediterranean Sea [41,42].

In Algeria, holothurians are exploited on a very small scale (mainly as fishing bait), but lately the existence of a nascent network of fishermen who process sea cucumbers into “bêche-de-mer” by themselves has been revealed [43,44,45,46].

In this study, four sea cucumber species (*H. poli, H. tubulosa, H. sanctori,* and *H. arguinensis*) were investigated to promote their potential as a new source for omega-3, omega-6, the production of bioactive peptides, and as functional food ingredients and nutraceuticals. The present study also focused on the evaluation of the in vitro potential of sea cucumber’s on living cells and for this purpose their hydrolysate cytocompatibility with primary human mesenchymal stem cells (MSCs) was tested. The preparation and physicochemical characterizations of different nanoliposomes made from sea cucumbers’ lecithin with or without HTF were carried out, to evaluate their ability as a natural carrier to deliver active substances into human MSCs cells.

## 2. Results and Discussion

### 2.1. Proximate Composition of the Body Wall

The proximate composition of the body wall is presented in Table 1. In fresh sea cucumbers, the body wall moisture varied from 61.85–67.76%, the highest value being recorded in *H. poli*. These contents were expected because most seafood contains a high level of water [47]. Ash content varied from 31.58–47.31% by dry weight, among which *H. arguinensis* showed the highest value. Ash content depends on the content of minerals like calcium, magnesium, iron, and zinc [48,49]. Crude protein ranged between 49.26–69.34% of dry weight; the highest value being obtained in *H. sanctori.* This result further indicated that our sea cucumbers could be developed as a food protein source. The total lipid, which varied between 2.57–4.30% of dry weight, was the lowest component. The highest lipid content was recorded in *H. poli*. Sea cucumbers are generally characterized by high protein and low lipid contents [50]. Sea cucumbers can therefore be considered in food formulations as ingredients with “high protein, low fat”.

Even though the species used in this study were different, the results were close to those of other sea cucumbers (Table 1) where moisture, ash, protein, and lipids ranged from 72.12–90.81%, 2.26–45.16%, 43.43–66.86%, and 0.76–5.66% respectively [34,51,52]. The proximate composition changes from one species to another, depending on many factors such as seasonal variations in feeding behavior and regional differences [53].

All species had a sufficient protein/lipid ratio (*H*. *poli*, 12:1; *H*. *tubulosa*, 13:1; *H*. *arguinensis*, 25:1; *H*. *sanctori*, 19:1), which is nutritionally significant considering the essential role proteins play in the human body.

### 2.2. Lipid Content and Composition

The lipid composition of the four holothurians is reported in Table 2. Total lipids showed a content of 3.55–6.43% of neutral lipids (NL) and 91.16–93.85% of polar lipids (PL), which consisted of 10.63–23.54% glycolipids and 69.02–80.22% phospholipids. The highest amounts of glycolipids and phospholipids were found in *H. poli* and *H. sanctori*.

In all samples analyzed, the phospholipids content was dominant and much higher than those obtained for other holothurian species like *Holothuria moebii*, *Holothuria impatiens*, *Stichopus chloronotus*, *Euaptagodeffroyi*, *Holothuria pardalis,* and *Apostichopus japonicus,* that ranged between 12.50–22.10% [54,55].

Most neutral lipids are used for energy storage [56]. In sea cucumbers, the neutral lipids obtained from phytoplanktonic sources are stored during the feeding period. When larvae are not able to obtain external nutriment sources, neutral lipids are degraded and used to fuel the rest of their metamorphosis during the larval development [57]. Phospholipids and glycolipids are the main component of algal lipids and in *Posidonia oceanica* leaves [58,59], which are one of the food sources for sea cucumbers [60,61].

### 2.3. Fatty Acids Composition

The obtained values varied among sea cucumbers (Table 3). All species showed a lower amount of monounsaturated fatty acids (MUFAs) compared to saturated fatty acids (SFAs), and polyunsaturated fatty acids (PUFAs), except for *H. tubulosa*, which showed a higher amount of MUFAs compared to SFAs. Lipid biochemistry in holothurians is affected by locations of sampling, food supply, feeding behavior and the kind of solvents used for lipid extraction [62,63].

The SFAs amount ranged between 12.21–32.38%, with the highest percentage found in *H. arguinensis.* The main SFAs found in the four species were palmitic acid (16:0) and stearic acid (18:0). *Holothuria tubulosa* showed the lowest percentage of palmitic acid (2.68%), while *H. arguinensis* had the highest percentage (14.60%), followed by *H. poli* and *H. sanctori* which showed almost the same percentages (4.60% and 4.15%, respectively). Similar results were found for stearic acid, *Holothuria arguinensis* also had the highest value (11.90%), followed by *H. poli* and *H. sanctori* which showed almost the same values (7.56% and 7.92% respectively), while *H. tubulosa* showed the lowest value (5.87%).

Total MUFAs detected in the holothurian species varied between 14.09–16.52%, with the highest amount found in *H. arguinensis*. The main MUFAs, found in the four species was *cis*-oleic acid (18:1n-9c). The *cis*-oleic acid showed almost the same amount between the four holothurians, that varied from 5.51–6.58%.

The PUFAs were found dominated in all the holothurian species and ranged between 50.90–71.80%, with the highest amount being found in *H. tubulosa*. The main PUFAs were arachidonic acid (ARA), α-linolenic acid (ALA) (18:3*n*-3), hexadecadienoic acid (16:2*n*-4), eicosapentaenoic acid (EPA) (20:5*n*-3), and docosahexaenoic acid (DHA) (22:6*n*-3). The highest content of ARA was detected in *H. tubulosa* (18.90%), followed by *H. poli, H. sanctori* and *H. arguinensis* (16.50%, 15.30%, and 10.50%, respectively). The highest amount of ALA was found in *H. poli* (11.0%), followed by *H. tubulosa*, *H. sanctori*, and *H. arguinensis* (10.0%, 9.33%, and 7.26%, respectively). *Holothuria tubulosa* showed the highest amount of hexadecadienoic acid (15.0%), followed by *H. poli*, *H. sanctori*, and *H. arguinensis* (12.6%, 11.2%, and 9.62%, respectively). EPA was comparatively higher than DHA in all the studied species. The highest amount of EPA was found in *H. tubulosa* and *H. sanctori*, which showed almost the same content (8.76% and 8.62%, respectively), followed by *H. poli* and *H. arguinensis* (7.90% and 5.07%, respectively). For DHA, *H. tubulosa* also showed the highest content (7.25%), followed by *H. poli* (6.56%), while *H. arguinensis* and *H. sanctori* contained almost the same amount (4.97% and 4.86%, respectively).

As known, sea cucumbers are deposit-feeders, that feed on bottom sediments consisting of detritus of vegetal and animal origin, phytoplankton, bacteria and diatoms mixed with sediments and also on the seabed soft sediments rich in EPA and DHA [60]. Sea cucumbers have a low metabolism and can’t synthesize long-chain fatty acids, but instead they accumulate them after consuming their feeding sources, which could influence their chemical composition, and their nutritional properties [64,65,66]. Palmitic acid, oleic acid, *cis*-oleic acid, and arachidonic acid are the main fatty acids found in the Mediterranean seaweeds (*Spyridia filamentosa*, *Acanthophora nayadiformis*, *Halymenia floresii*, *Cystoseira corniculate, Padina pavonia* and *Stypopodium schimperii*) [67,68]. Palmitic acid and stearic acid were also the main fatty acids found in the Mediterranean microalgae *Chlorella sp.* [69]. α-Linolenic acid is an essential fatty acid present in vegetable oils, a precursor of the long-chainn-3 fatty acids EPA and DHA, and is usually found in high amounts in the leaves of the Mediterranean seagrass *Posidonia oceanica* [70], whose detritus are exploited as a food source by these holothurians [61]. Diatoms and dinoflagellates are another source of EPA and DHA, respectively [71]. Hexadecadienoic acid is usually found in marine species such as, the marine fungus *Clonostachys rosea* [72], and the Mediterranean sponge *Chondrilla nuclear* [73], which we assume could be another food source for the sea cucumbers. Even though, sea cucumbers’ food sources are mainly marine, some physical parameters of the environment such as terrestrial inputs can also influence their nutritional properties [71].

Omega-3 fatty acids such as ALA, ARA, EPA and DHA have been shown to be effective in the prevention and treatment of several diseases. ALA is used in both dietary and pharmaceutical forms as a supplement to minimize the risk of stroke and as a nutraceutical to improve brain resistance to stroke damage [74]. ARA plays a crucial role in maintenance of cell and organelle integrity, vascular permeability, and cellular signaling [75]. These properties might explain ARA′s critical role in neuron function, brain synaptic plasticity, and long-term potentiation in the hippocampus [48,49]. It has been demonstrated that higher intakes of EPA and DHA, decrease artery coronary heart disease, prevent cardiac arrhythmias, high blood pressure, have anti-thrombotic activity, inhibit prostaglandin, and are important for the visual and neurological functions [76,77,78].

The ratio of *n*-6/*n*-3 is very important and a balanced ratio should be applied when formulating balanced diets, due to the antagonistic effects of omega-6 and omega-3 [79]. A ratio of less than 10 is generally recommended [80]. The FAO recommends a ratio between 5:2 and 8:1 [81]. The *n*-6/*n*-3 ratio of the four sea cucumbers collected from the Algerian coast was in the range of 0.67–1.03, which indicates that they are safe for consumption.

### 2.4. Differential Scanning Calorimetry of Lipids (DSC)

The oils of our sea cucumber are characterized by a significant amount of polyunsaturated *n*-3 fatty acids, which have increasing importance as nutraceuticals. The melting points and enthalpies of the four sea cucumbers oils are shown in Table 4. Oils melt over a temperature range and do not have a specific melting temperature. The changes in the melting process may not be visible, but can be measured by a DSC instrument, and often exhibit multiple endotherms [82]. DSC was applied to determine the thermal, oxidative stability, and the quality of sea cucumber oils, as well as their possible interactions during the formulation process if ever used as nutraceuticals. The melting curves for the four sea cucumbers oils showed one endothermic peak at different heating rates at −41.71, 2.41, −33.33, and −2.56 °C for *H. poli*, *H. tubulosa*, *H. arguinensis,* and *H. sanctori*, respectively. The melting temperatures of sea cucumber oils differed from other fish oils processed from sardine (−22.60 °C), cod liver (−16.53 °C), and salmon (−6.80 °C) [83]. The enthalpies of fusion (∆H) ranged between 0.81–5.79 J/g, and differed from those found for sardine, cod liver and salmon oils (1.43–15.34 J/g) [83]. The melting temperatures of the oils differed between the species. Since all experiments were performed in the same DSC instrument, and under the same conditions, the discussion will be focused on the changes of the melting profiles of sea cucumber’s oils in terms of their triacylglycerol (TAGs) compositions [82].

Generally, highly trisaturated TAGs (SSS) melt at higher temperatures when compared with highly tri-unsaturated TAGs (UUUs); while the mono-unsaturated (SSUs) and di-unsaturated TAGs (SUUs) melt in between these two groups [82]. For our results, we assume that the lower-temperature endotherm of *H. poli*, *H. arguinensis*, and *H. sanctori* might correspond to the melting range of highly unsaturated TAGs (UUU), while the higher-temperature endotherm of *H. tubulosa*, could be due to the melting of a group of UUUs and SUUs [82].

The differential scanning calorimetry (DSC) curves could also be markedly influenced by the degree of saturation or unsaturation of fatty acids [83]. The melting points of saturated fatty acids increase with increasing chain length, possibly due to the intermolecular dispersion force which increases with the increased number of carbons in chains [84]. In the case of unsaturated fatty acids, the increased number of double bonds decreases the melting point. The low melting temperature and enthalpies of fusion indicated that the lipids contained a high proportion of unsaturated fatty acids [85].

As mentioned before, all the heating curves showed only one endotherm peak. The number of peaks in the heating scans is proportional to the heating rate. The changes in DSC profile with heating rate are complicated, and the curves with higher rates show a single endotherm peak. The presence of a single endothermic peak during the entire melting phase, can also indicates that sea cucumber oils remain stable throughout the heating process.

Considering the complexity of the DSC melting behavior of oils, thermal behavior interpretations based on such DSC scans, the identification, and interpretation of their thermal events must be made with caution. To the best of our knowledge, these thermal events have not been reported before for the sea cucumber oils used in the present study.

### 2.5. Fourier-transform Infrared (FTIR) Spectroscopy

Fourier-transform infrared (FTIR) spectroscopy provides important information about the conformation and functional groups of polysaccharides. Table 5 lists the FTIR spectra bands for the four sea cucumber oils, in the wavenumber range of 4000–400 cm^−1^. Since that was no previous data of the infrared spectra (IR) of Mediterranean sea cucumber oils, the bands were identified and assigned to specified molecular groups on the basis of previous studies using biochemical standards.

Our results showed that the sea cucumbers’ spectra were complex, with several peaks arising from the contribution of different functional groups belonging to their lipids (Table 5). Although the investigated oils’ bands seemed similar, there are considerable differences in the intensity of relevant bands as well as in their precise frequency, clearly related to the composition of the oils [86]. The bands appearing at 2925–2920 cm^−1^, and 2854–2850 cm^−1^ were assigned to the antisymmetric vibration in CH_2_ groups, and symmetric stretching in the CH_2_ groups of alkyl chains [87,88], with both bands showing a variation in absorbance between the four species, *H. poli* showed the highest. The two bands are usually used to monitor the thermotropic phase transition of alkyl chains in phospholipids and the changes in the frequency and bandwidth of the CH_2_ stretching vibrations are directly related to the conformational order of lipid alkyl chains [89,90]. The stretching vibrations of the carbonyl C=O ester groups in triacylglycerols were detected at 1739–1614 cm^−1^ [91,92]. A decrease in the absorbance was visible for *H*. *tubulosa*, *H*. *arguinensis* and *H*. *sanctori,* which suggested a decreased concentration of the ester groups belonging to triacylglycerols and the appearance of aldehydes and ketones, the secondary oxidation products of the degradation of hydroperoxides [93,94].

The bands at 1471–1440 cm^−1^ correspond to scissoring vibrations of CH_2_ groups, while CH_2_ bending (rocking) vibrations were detected at 756–752 cm^−1^ [94,95,96], *Holothuria poli* showed the lowest and the highest absorbance, respectively. The late vibrations bands provide information on alkyl chain packing. In particular, all*-trans* configuration alkyl chains, pack in a solid lattice, give rise to factor group splitting of both the CH_2_ scissoring and rocking bands [97]. The rocking vibrations of =C–H *(cis)* appeared at 1411–1400 cm^−1^ and –HC=CH– (*cis*-) bending out of plane bands, appeared at 931–902 cm^−1^ [88,94,98]. The highest absorbance was found in *H. tubulosa*. The two bands are usually used to characterize the vibrations of *cis*-unsaturated structures [99]. In all four sea cucumbers, the bands at 1163 cm^−1^ were associated with CH_2_ out-of-plane deformation modes and major peaks at 1024–1004 cm^−1^, represented the symmetrical-C–O–C stretching [91], with *H. poli* showing the highest absorbance. Both bands are primarily from triacylglycerols, phospholipids, and cholesterol esters [100].

The FTIR spectra provided a molecular characterization of the sea cucumbers’ oils. FTIR spectra identified three lipid classes present in the oil of sea cucumbers, namely triacylglycerols, phospholipids, and cholesterol. Similar IR spectra were found for several fish oils from tuna (*Katsuwonas pelamis*, *Thunnus albacares*, *Thunnus alalonga, Thunnus obesus*), bonito (*Sarda orientalis),* fin-fish hoki (*Macruronus novaezelandiae*), school sharks (*Galeorhinus galeus*), rig sharks (*Mustelus antarcticus*), and spiny dogfish (*Squalus acanthias*) [101].

### 2.6. Phospholipid Composition

The results presented in Table 6 are the polar lipids fraction composition for the studied sea cucumbers. The phospholipid (PL) percentage ranged from 55.20–69.85% (of total lipids). Similar values were found in other seafoods known for their high levels of phospholipids such as shrimps (56–69%) [102,103,104], mussels (57–67%) [105,106,107], oyster (50%) [108], squid (64–67%)[109,110,111,112]. PL ′s normal dietary intake is 2–8 g a day, representing 1–10% daily fat intake [113], which makes our sea cucumbers a valuable source of marine phospholipids. The five phospholipids found in the sea cucumbers were cardiolipin (CL), phosphatidylglycerol (PG), phosphatidylcholine (PC), phosphatidylethanolamine (PE), and phosphatidylserine (PS).

The amount of phospholipids differed between the four species (Table 6). The highest percentage of CL, which ranged between 5.13–11.25% of total phospholipids, was found in *H. sanctori*. CL plays important role in cellular processes and pathways that are crucial for heart function, including mitochondrial function, mitochondrial protein import, autophagy/mitophagy, and the protein kinase C pathway [114]. PG amount varied between 9.10–14.38%, the highest amount was found in *H. poli* and *H. arguinensis*. Studies showed that PG can protect human retinal pigment epithelial cells against apoptosis and also stimulate keratinocyte [115,116]. PC ranged between 51.48–58.57%, the highest value was found in *H*. *tubulosa* and *H*. *arguinensis* (Table 6). PC is essential for the neurotransmitter acetylcholine synthesis, can nourish the brain and improve intelligence [117]. PE and PS ranged between 6.31–8.10% and 0.91–6.47%, respectively (Table 6). PE is known for playing an important role in membrane fusion [118]. PS improves nerve cells function, regulate nerve impulse conduction, enhance the memory, and is featured in the apoptosis [119,120,121].

This is, to the best of our knowledge, the first study documenting the phospholipids profile of Mediterranean sea cucumbers. Most researches in this respect were carried out for different species such as the Asian sea cucumber *Apostichopus japonicus* [66].

### 2.7. Enzymatic Hydrolysis

**Figure 1:**
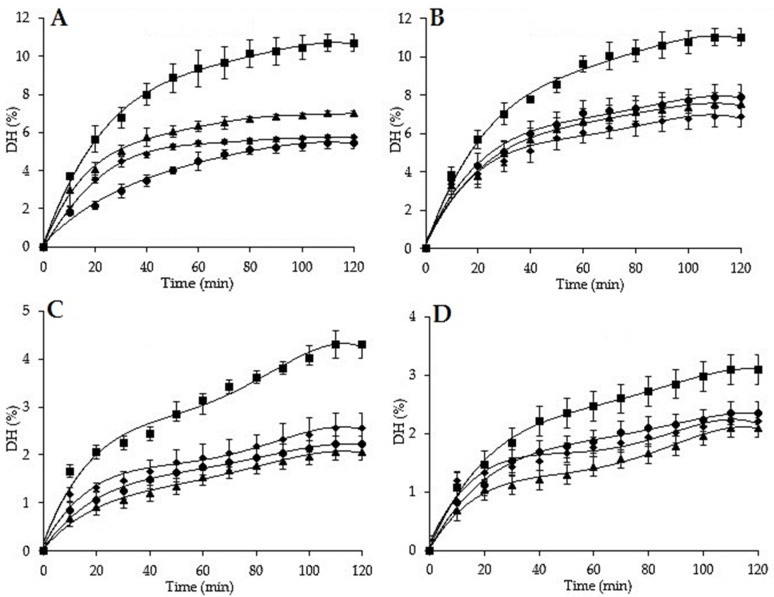
Degree of hydrolysis of *H. poli*, *H. tubulosa*, *H. arguinensis*, and *H. sanctori*, using Alcalase 2.4 L and Neutrase 0.8 L at 2% and 5%. The error bars represent standard deviation of means ± SD of at least three hydrolysis (*n* = 3) for each sea cucumber and at each enzyme concentration. (**A**): Degree of hydrolysis with Alcalase 2.4L at 2%; (**B**): Degree of hydrolysis with Alcalase 2.4L at 5%; (**C**): Degree of hydrolysis with Neutrase 0.8 L at 2%; (**D**): Degree of hydrolysis with Neutrase 0.8 L at 5%.

The sea cucumbers’ body walls were hydrolyzed successfully with two proteases (Alcalase 2.4 L and Neutrase 0.8 L) at 2 and 5%. The total histories of the course of the hydrolysis and the degree of hydrolysis (DH) values of each studied sea cucumber are presented in Figure 1. The enzymatic hydrolyzes were characterized by three distinct stages. During the first 60 min, the DH values increased rapidly, suggesting multiple peptide bonds being cleaved [122,123]. Then, the hydrolysis rate decreased slightly until it reached a plateau around 100 min. This could be explained by the decrease in the available hydrolysis sites, or the absence of the enzyme specific amino acids (AAs) [124,125]. After that, the hydrolysis reactions followed a linear trend until the end of each experiment, the result of either a limitation on the available cutting sites, enzyme denaturation and/or product inhibition [74]. These results were similar to those reported for *Stichopus horrens* [123], *Actinopyga lecanora* [126], and *Isostichopus badionotus* [17].

All the proteolysis curves showed that all the studied sea cucumbers are degradable by the two proteases, but Alcalase at 5% showed the most effective enzymatic hydrolysis compared to the other reactions (Figure 1), with the highest DH values of 7.92%, 11.01%, 7.52%, and 6.87%, respectively for *H. poli*, *H. tubulosa*, *H. arguinensis*, and *H. sanctori*, indicating that Alcalase cleaved more peptide bonds than Neutrase, which implied that there were more available cutting sites for Alcalase than for Neutrase. These results can be also explained by the fact that Alcalase has a broad specificity substrate, a broad working pH and temperature range can hydrolyze both native and denatured proteins, and is stable against autoproteolysis [127].

### 2.8. Angiotensin-Converting Enzyme Inhibition Activity (ACE) and IC_50_

To study the changes of the half maximal inhibitory concentration (IC_50_) among the four sea cucumbers species, the ACE inhibitory activities of their hydrolysate’s fractions were measured. Sea cucumbers were hydrolyzed using two different proteases for 120 min (Figure 1). Hydrolyzed samples were collected at the end of the proteolysis. Those with the highest DH were analyzed for ACE Inhibition Activities and IC_50_ determination (Alcalase 5%, <1 kDa). Sea cucumber hydrolysates produced with Alcalase showed considerable ACE inhibitory activities varying over a wide range of inhibitory concentration (IC_50_) from 0.30 to 0.51 mg/mL (Table 7). These values reflected hydrolysis effectiveness enhancing ACE inhibitory [123]. Among the tested sea cucumber hydrolysates, *H. sanctori* exhibited the lowest IC_50_ value, indicating the highest ACE inhibitory activity. The variation in IC_50_ among the four sea cucumber species can be attributed to the impact of enzyme specificity, which is a key factor influencing both the characteristics of hydrolysates and thus the nature and composition of the peptides produced [128]. Alcalase is known for its specificity mainly for hydrophobic amino acids while Neutrase has specificity mainly for leucine and phenylalanine [129]. Alcalase tends to produce peptides whose C-terminals are amino acids with large side chains and no charge (aromatic and aliphatic amino acids), such as tyrosine, phenylalanine, tryptophan, methionine, valine, leucine and isoleucine. Alcalase cleaves the peptide bond of aliphatic or aromatic amino acids [130], leading to the formation of new peptides with a high content of hydrophobic amino acids [123]. It was demonstrated that peptides containing hydrophobic (aromatic or branched side chains) amino acid residues possess a high inhibitory effect [131], like naturally occurring ACE-inhibitory peptides, highly rich in hydrophobic amino acids [132,133]. However, the comparison of the present results with the other studies is difficult due to: (1) the absence of literature on the ACE inhibitory activity of *H. poli*, *H. tubulosa*, *H. arguinensis,* and *H. sanctori*; (2) the variations in the species; (3) the proteolysis conditions (temperature; time…etc.) and (4) the choice of enzyme and its concentration. However, the IC_50_ values of (*H. tubulosa*, *H. arguinensis* and *H. sanctori*) were quite close to those found for *Acaudina molpadiodea* gelatin, hydrolyzed with Alcalase and bromelain (1 kDa, 0.35 mg/mL) [134]. *Holothuria poli* showed the same results as those obtained for *Parastichopus californicus* collagen hydrolysate digested by pepsin (0.51 mg/mL for 3 h) [135]. Our results were lower than those reported for other sea cucumbers that have been hydrolyzed with Alcalase like *Actinopyga lecanora* (1.50 mg/mL after 8 h) [126], and *Stichopus horrens* (0.615 mg/mL after 5 h) [123]. On the other hand, IC_50_ values for the alcalase hydrolysate were higher than those reported for boiled *Isostichopus badionotus* hydrolyzed sequentially with pepsin and Corolase PP under conditions simulating gastrointestinal digestion, the three hydrolyzed fractions (3 kDa, >3 kDa, and <3 kDa) showed respectively IC_50_ values of 0.135 mg/mL, 0.120 mg/mL and 0.038 mg/mL, suggesting that double digestion (Pepsin+Corolase PP) contributed to the increase of ACE inhibitory activity [136].

The ACE inhibitory activity and proteolysis are influenced by the protein structure, and the enzyme specificity that lead to the cleavage of different bonds and to the creation of peptides with differing *N*-and *C*-terminal [123].

### 2.9. Liposome Size and Potential Zeta Measurements

The particle sizes of different nanoliposomes with and without hydrolysate were measured immediately after sonication. The hydrodynamic diameter of nanoliposomes for sea cucumbers were 169 nm with 36 mV for Lip HPF empty, 188 nm and −35 mV for Lip HPF 10 mg/mL; Lip HAF empty 154 nm with −28 mV and Lip HAF 10 mg/mL 188 nm with −30 mV; Lip HTF empty 162 nm with −28 mV and 163 nm with −30 mV for Lip HTF 10 mg/mL; Lip HSF empty 142 nm with −30 mV and Lip HSF 10 mg/mL 117nm with −32 mV. The size of nanoparticle increases after the encapsulation of hydrolysate.

### 2.10. Cytocompatibility of Hydrolysates

**Figure 2:**
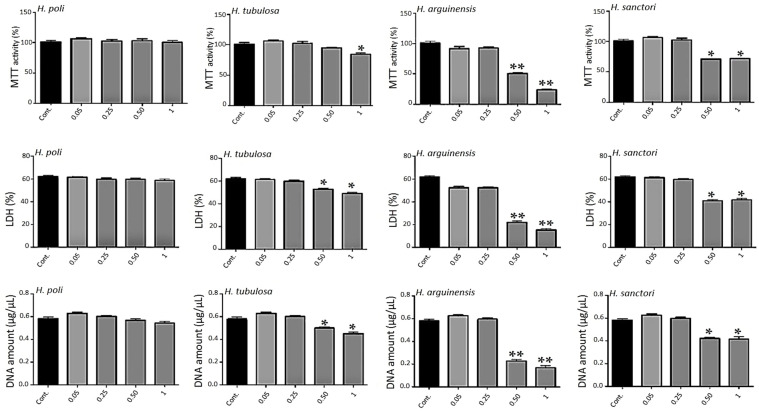
Impact of hydrolysates on metabolic activity. Human MSCs were exposed to increasing concentrations of hydrolysates (0.05, 0.25, 0.5 and 1 mg/mL) for 7 days. Metabolic activity was assessed using the 3-(4,5-dimethylthiazol-2-yl)-2,5-diphenyltetrazolium) (MTT) assay. Lactate Dehydrogenase (LDH) release was determined as described under the Materials and Methods section. DNA concentrations were measured to estimate the proliferation of the cells. For each tested condition, the cell metabolic activity results are presented in% versus control (Cont.) condition (100%). The error bars represent standard deviation of means ± SD of at least three individual experiments. * *p* < 0.01, ** *p* < 0.001 compared to control for each time point.

Experiments were realized with the alcalase 5% fraction of hydrolysates from *H. poli*, *H. tubulosa*, *H. arguinensis*, and *H. sanctori*. The data on Figure 2 showed a 7-days treatment as no large modifications were observed for shorter times (3 days and 5 days, not shown).

The metabolic activity was based on the ability of the living cells to reduce tetrazolium salt of 3-(4,5-dimethylthiazol-2-yl)-2,5-diphenyltetrazolium) (MTT) into formazan crystals. All conditions showed an important metabolic activity (Figure 2) for all concentrations for *H. poli* and *H. tubulosa*, respectively. For *H. arguinensis* and *H. sanctori*, the high concentration of 0.5 and 1 mg/mL had a strong impact on metabolic activity (Figure 2).

The cytotoxicity of hydrolysates was evaluated after 3, 5 and 7 days after exposure to various concentrations by a lactate dehydrogenase (LDH) assay (Figure 2, day 7 only). Cells not exposed to hydrolysates were considered as control. There was no statistically significant cytotoxicity difference between control and all the concentrations of *H. poli* (Figure 2) and no difference in proliferation (Figure 2). For the other 3 sea cucumbers, only the highest ones, 0.5 and 1 mg/mL appeared to be cytotoxic for human mesenchymal stem cells (MSCs) (Figure 2). Again, the two extreme concentrations triggered a decrease in proliferation as assessed by DNA quantification (Figure 2).

**Figure 3:**
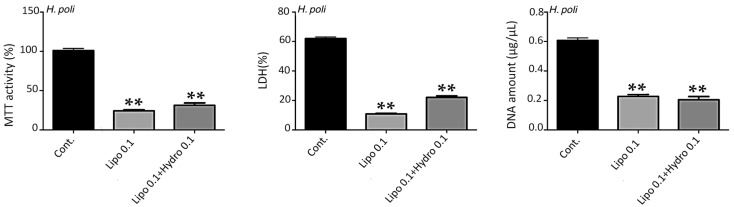

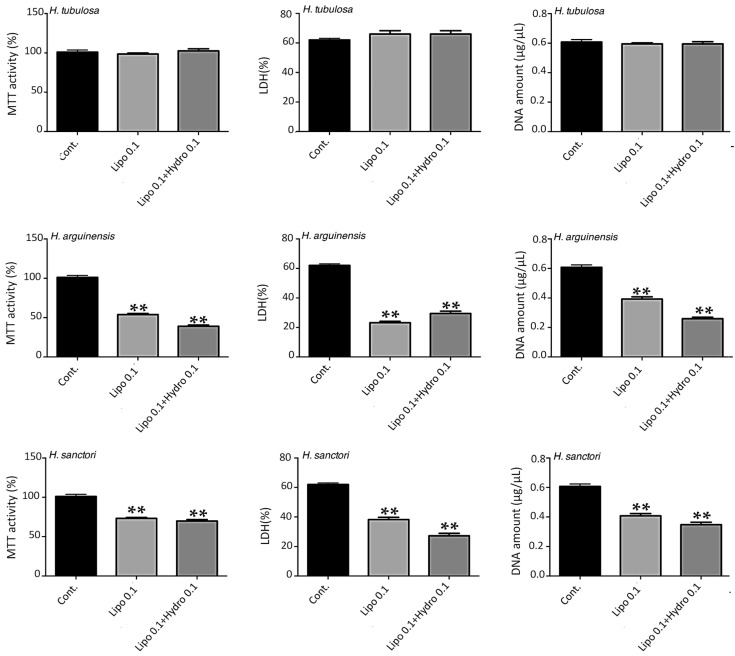
Impact of nanoliposomes without hydrolysates (Lipo 0.1) and with hydrolysates (Lipo 0.1+ Hydro 0.1) on human MSC. Human MSCs were exposed to nanoliposomes alone (0.1 mg/mL) or hydrolysates in nanoliposomes (0.1 mg/mL) for 7 days. Metabolic activity was assessed using an MTT assay, LDH release was determined as described under the Materials and Methods section, DNA concentrations were measured to estimate the proliferation of the cells. The results shown for *H. poli*, *H. tubulosa*, *H. arguinensis*, and *H. sanctori* are mean ± SD of at least three individual experiments. ** *p* < 0.001 compared to control for each point. The control condition for MSC metabolic activity was used as the reference value.

The nanostructures alone (i.e., empty nanoliposomes or nanoliposomes associated with HTF) (Figure 3) showed no modification of metabolic activity or proliferation and no cytotoxicity whatever the concentrations (0.01, 0.05, 0.10 and 0.50 mg/mL) and the time of stimulation (1, 3, 5 or 7 days, only 7 days on Figure 3). This demonstrates the harmlessness of the tested conditions for HTF.

For the other hydrolysates, i.e., HAF, HSF and HPF (Figure 3) the concentration of 0.1 mg/mL of liposomes was toxic, while 0.05 mg/mL hydrolysates were more harmless (data not shown).

## 3. Materials and Methods

### 3.1. Chemicals

Chloroform, acetone, and acetonitrile (CAN) were purchased from Biosolve (Dieuze, France), methanol was obtained from Carlo Erba Reagent (Carlo Erba Reagent, Val-de-Reuil, France), ammonia was purchased from EMSURE (Fontnay-Sous-Bois, France), trifluoroacetic acid (TFA) was supplied by Thermo Fisher Scientific (Waltham, MA, USA). Alcalase^®^ 2.4 L from *Bacillus licheniformis*, Neutrase^®^ 0.8 L from *Bacillus amyloliquefaciens*, hexane, boron trifluoride-methanol solution, hippuryl histidyl-leucine (HHL), 2-(cyclohexylamino)ethanesulfonic acid (CHES), (ethylenediamino) tetraacetic acid (EDTA), angiotensin converting enzyme (ACE) derived from rabbit lung, C23, and all HPLC standards were purchased from Sigma-Aldrich (Munich, Germany). All other reagents used in this study were of analytical grade.

### 3.2. Samples Collection

Four species of sea cucumbers (*Holothuria poli, Holothuria tubulosa, Holothuria arguinensis,* and *Holothuria sanctori*), were collected from three sampling sites on the west coast of Algeria [Oran harbor (35°42′34.2″N, 0°39′20.8″W), Kristel (35°49′22.3″N, 0°29′26.6″W), and Falcon Cap (35°46′21.5″N, 0°47′51.0″W)], in October 2018. Immediately after collection, sea cucumbers were kept separately in zip-lock plastic bags, frozen at −20 °C, and then were transferred to the “Laboratoire d’Ingénierie des Biomolécules” (LIBio), Lorraine University, France), using an icebox. Sea cucumbers samples were left to thaw slowly at room temperature, and the body wall was separated from the viscera (all internal organs) and was thoroughly washed with distilled water to remove sand and dirt. Although, there are many recommendations concerning the handling of sea cucumbers, no ethical code has been established. In our study, we were careful to ensure that all specimens were treated with respect and empathy, using an ethically responsible research.

### 3.3. Proximate Composition of Sea Cucumber’s Body Wall

Moisture content was measured according to the standard method AOAC [137], the body wall was dried in an oven at 103 °C for 6 h, until constant mass. Ash content was estimated according to the standard method AOAC [138], by incineration of the dry body wall in a muffle furnace at 600 °C for 12 h. The protein content was determined according to the Kjeldahl method [139], a conversion factor of 6.25 was used to convert the total nitrogen into crude protein. Lipids were extracted according to Folch et al. [140] modified by Christie [141]. Results were expressed as a percentage obtained from triplicate analyses.

### 3.4. Determination of the Lipid Classes by Iatroscan

The lipid classes of sea cucumbers were determined using an Iatroscan MK-5 TLC-FID (SES GmbH, Bechenheim, Germany). The analysis was done according to the protocol described by Hasan et al. [85]. To determine the proportion of neutral and polar lipid fractions, two different migrations were used. The lipids migrate according to their affinity for both eluents. The first migration allows the separation of apolar compounds (triacylglycerols: TAG) and polar compounds (phospholipids and glycolipids), using a solvent mixture (hexane: diethyl ether, 70:30, 20 min migration time). The second migration allows the separation of the polar compounds (phospholipids and glycolipids), using a second solvent mixture (chloroform:methanol:water:ammonia, 65:35:5:0.28, 40 min migration time). Chromstar software was used to provide peaks representative of sample composition; the polar compounds were separated by their retention time. Area percentages were presented as the mean value of three repetitions analyses.

### 3.5. Fatty Acid Composition

Fatty acid methyl esters (FAMEs) from sea cucumbers lipids, were prepared according to the method described by Ackman [142], using a 14% boron trifluoride-methanol solution (BF_3_/MetOH), as the esterification reagent. The FAMEs were then analyzed using a 2010 gas chromatography system (GC, Shimadzu, Kyoto, Japan), equipped with a flame ionization detector. Separation of FAME was carried out on a fused silica capillary column (60 m, 0.25 mm i.d. 0.20 mm, l m thicknesses). Injector and detector temperatures were settled at 250 °C. The column temperature was fixed initially at 120 °C for 3 min, then raised to 180 °C at a rate of 2 °C min^−1^ and maintained at 220 °C for 25 min. Individual fatty acids were identified using tricosanoic acid as standard. Peak integration is done on GC software. The results were obtained from triplicate analyses.

### 3.6. Thermal Analysis of Oils by Differential Scanning Calorimetry (DSC)

Differential thermal analysis (DSC), was done to determine the thermal stability of sea cucumber lipids according to Hasan et al. [85]. The thermal transition of the lipids was measured using a calibrated differential scanning calorimeter (Pyris model, TA Instrument, New Castle, DE, USA). The apparatus was calibrated before by measuring the melting temperature and the enthalpy of indium (mp: 156.6 °C., H: 28.45 J/g). The sample (10 mg) were weighed into aluminum pans, sealed tightly, and then placed in the calorimeter. The program consists of gradually warming the samples from −80 °C up to 80 °C (5 °C min^−1^). Temperature data variation with different heat flows, corresponding to peak maxima and melting enthalpies (J/g) were calculated by the TA Instruments Analysis Software.

### 3.7. Fourier-Transform Infrared (FTIR) Spectroscopy

FTIR spectra were recorded using a Tensor 27 mid-FTIR spectrometer (Bruker, Billerica, MA, USA), equipped with a diamond ATR module and a DTGS detector according to Hasan et al. [85]. Scanning rate was fixed to 20 kHz and 128 scans were performed for both reference and samples between 400 cm^−1^ and 4000 cm^−1^ at a resolution of 2 cm^−1^ at room temperature. An initial reference spectrum was then recorded. Next, a small amount of each sample was put on the diamond crystal of the optical cell and a minimum of two separate experiments were done for each sample. Also, all treatments were carried out using the OPUS software (Cooperative Library Network Berlin-Brandenburg, Stuttgart, Germany). Crude absorbance spectra were smoothed using a nine-points Savitsky–Golay smoothing function. Then spectra were centered and normalized using OPUS software.

### 3.8. Purification of Phospholipids by Acetone Precipitation

To purify sea cucumber phospholipids, a modified acetone precipitation method was used [85,143,144]. In 2 mL of chloroform, 1.3 g of lipids were dissolved, then emptied into 10 mL of acetone (approximate ratio of 1:7.7) [143], under vigorous stirring at ambient temperature. The solutions were kept overnight at −18 °C, to allow phospholipids precipitation, then centrifuged at 1000 rpm. The precipitates were redissolved in chloroform, and the purification procedure was repeated once again. The final precipitates (purified phospholipid) were dried under nitrogen for 1 h. The residues of acetone and chloroform were further removed under vacuum at 40 °C.

### 3.9. Separation and Quantification of Phospholipid Classes by HPLC

A Surveyor HPLC system (Thermo Fisher Scientific, Waltham, MA, USA) was used for the separation and quantification of different classes of phospholipids according to Stith et al. [145] with some modifications made by according to Hasan et al. [85]. The analyses were carried out on a Phenomenex silica column. The phospholipid classes were detected using an evaporative light-scattering detector (ELSDLT II). Chromeleon^®^ software controlled the gradient and injection system. The column was maintained at 50 °C in the column heater. The injection volume was set to 15 μL, and the acquisition time was 65 min per sample. The phospholipids were separated using a gradient of three solvents; the gradient had a constant flow 1 mL min^−1^, with solvent A: chloroform-methanol −25% ammonium hydroxide (80:19:1), B: chloroform-methanol −25% ammonium hydroxide (60:39:1), and C: chloroform-methanol-water −25% ammonium hydroxide (60:34:5:1). Gradient time table (A%/B%/C%, *v*/*v*/*v*): from 0 to 5 min 100/0/0, from 5 to 25 min 0/100/0, from 25 to 35 min 0/0/100, at 50 min 0/0/100, at 55 min 100/0/0, and finally at 65 min 100/0/0.

The phospholipid classes were identified and quantified by comparing their retention times with those present in standard calibration curves. Different volumes of the standard were used for the calibration curves to have different concentrations. Area percentages were presented as the mean value of three repetitions analyses.

### 3.10. Preparation of Enzymatic Hydrolysates

The hydrolysis experiments were carried out in duplicate according to the pH-stat procedure as described by Adler-Nissen and Gbogouri et al. [146,147], with some modifications. Before the enzymatic hydrolysis, the freeze-dried body walls were ground into small pieces. For each experiment, 10 g of sea cucumber sample was mixed with 100 mL distilled water, and then hydrolyzed independently with each enzyme at the optimal conditions specific to each one (pH, temperature): Alcalase 2.4 L (pH 8, 55 °C), and Neutrase 0.8 L (pH 7.5, 50 °C) at 2% (0.1–0.3 mL of enzyme, depending on the protein levels of the each sample), and 5% (0.25–0.35 mL enzyme, depending on the protein levels of the each sample). Proteolysis was carried out for 120 min in a water-bath with continuous stirring at 450 rpm. The enzymatic reactions were immediately stopped by heating at 90 °C for 10 min to inactivate the proteases, followed by cooling at room temperature. The resulting hydrolysates were centrifuged at 6000*×* g for 20 min at 15 °C, to separate insoluble and soluble fractions. Finally, the soluble phase recovered was frozen at −20 °C, until further use.

### 3.11. Degree of Hydrolysis

The degree of hydrolysis (DH) is the percentage ratio between the number of peptide bonds cleaved and the total number of bonds available for proteolytic hydrolysis. In this work, the DH was obtained according to Adler-Nissen [146] using Equation (1):

(1) $$\mathrm{DH}\left(\%\right)=\frac{N_{B}\times B}{\mathrm{MP}\times \alpha \;\times {\;h}_{\mathrm{tot}}}\times 100$$

where B: volume of added NaOH (mL); N_B_: normality of NaOH; α: the mean degree of dissociation of alpha-amino groups; MP: protein mass (NT × 6.25) present in the reaction medium (g); h_tot_: number of peptide bonds in the protein for sea cucumbers (4.33 meq/g).

The degree of dissociation is defined as Equation (2):

(2) $$\alpha =\frac{10^{\mathrm{pH}-\mathrm{pK}}}{1+10^{\mathrm{pH}-\mathrm{pK}}}$$

where pK represents the average α-amino functions released during hydrolysis(Equation (3)):

(3) $$\mathrm{pK}=7.8+\left(\frac{298-T}{298\times T}\right)\times 2400$$

where T is the temperature expressed in Kelvin. The results were obtained from triplicate analyses.

### 3.12. Hydrolysate Purification by Ultrafiltration

The hydrolysates with the highest hydrolysis degree were fractionated by ultrafiltration using a peristaltic pump (80 rpm, 551 mL min^−1^, 2 bars). The hydrolysates were dissolved in distilled water until a volume of 400 mL. Two molecular weight (MW) cut-off membranes were used (>10 kDa, and then <1 kDa). The final purified fractions were frozen at −18 °C, and then freeze-dried for 72 h.

### 3.13. ACE Inhibitory Activity Assay

The ACE inhibitory activity was quantified for the most promising hydrolysate and fractions (highest hydrolysis degree) according to Cushman et al. [148]. ACE mixture assay contained 50 μL sample (4.3 mg in 2 mL buffer CHES 50 mM, NaCl 300 Mm, pH 8.3), 20 μL ACE (0.1 U ACE rabbit lung in 200 μL of buffer: CHES 50 mM, NaCl 300 mM, glycerol 5%, pH 8.3), and 120 μL HHL (22.23 mg HHL in 6 mL buffer CHES 50 mM and NaCl 300 mm, pH 8.3). The mixture was incubated at an optimal temperature of 37 °C for 45 min, then the reaction was inactivated using 75μL of the STOP solution (15 µM captopril, 3 mM EDTA and 0.2% TFA). The solution was vortexed and then filtered through a 0.22 μm syringe filter. A quantity of 50 μL of the sample is injected onto an HPLC column (Altima^®^ C18 150 × 2.1) (Shimadzu LC-10) using the following method: gradient at a constant flow of 0.2 mL min^−1^, with solvent A: ultra-pure water 0.1% TFA (100:0.1), B: ACN-0.1% TFA (100:0.1). Gradient timetable (A%/B%, *v*/*v*): at 0 min 87/13, at 7 min 50/50, at 17 min 1/99, at 18 min 87/13, at and 35 min 87/13. The column temperature was maintained at 29 °C.

### 3.14. IC_50_ Determination of the Hydrolysates

The IC_50_ value is defined as the concentration of hydrolysates required to inhibit the 50% of ACE activity under experimental condition. The IC_50_ of the different hydrolysates was determined by plotting the ACE inhibition (%) activities against the various concentrations of hydrolysates. The results were obtained from triplicate analyses.

### 3.15. Liposome Preparation

Nanoliposomes were prepared according to the method of Bouarab et al. [149] with some modifications. Sixty mg of sea cucumber lecithin was added to 2.94 mL of distilled water and the suspension was agitated for 4 h under nitrogen in order to obtain nanoliposomes with 2% lecithin. For the nanoliposomes containing protein hydrolysate, 30 mg/mL of hydrolysate at UF 1 kDa was used for obtaining a final concentration of hydrolysate at 10 mg/mL. The samples were then sonicated at 40 kHz and 30% of full power for 4 min (1s on, 1s off) to obtain a homogeneous solution. Liposome samples were stored in a glass bottle in the dark at 37 °C.

### 3.16. Liposome Size and Potential Zeta Measurements

The liposome size was analyzed by dynamic light scattering (DLS) using a Malvern Zetasizer Nano ZS (Malvern Instruments, Malvern, UK). The used protocol was adapted from Hasan et al. [150]. The samples were diluted (1:500) with ultra-filtrate distilled water and placed in vertical cylindrical cells (10 mm-diameter). The scattering intensity was measured at a scattering angle of 173° relative to the source using an avalanche photodiodes detector at 25 °C. The refractive index (RI) and absorbance were fixed respectively at 1.471 and 0.010 at 25 °C. The measurements were performed in five repetitions.

### 3.17. Cytocompatibility Assays

To evaluate the impact of hydrolysates of Mediterranean sea cucumber species (*Holothuria poli*, *Holothuria tubulosa*, *Holothuria arguinensis,* and *Holothuria sanctori)* (HPF, HTF, HAF and HSF respectively) on cell behavior, different parameters were estimated: system potential cytotoxicity, cell metabolic activity, and cell proliferation.

### 3.18. Cytotoxicity Assays

The cytotoxicity test was performed after 3, 5 and 7 days using the Cytotoxicity Detection Kit^PLUS^ (LDH) (#04744926001; Roche, Saint Louis, MO, USA) according to the manufacturer’s instructions. This assay is based on the measurement of lactacte deshydrogenase (LDH) activity released from the cytosol of damaged cells. Three controls are included: background control (assay medium), low control (untreated cells corresponding to the control condition) and high control (a positive control where a maximum of LDH is released due to cell lysis). The absorbance was read on a spectrophotometer at 490 nm (Varioskan^®^ Flash, Thermo Fisher Scientific, Waltham, MA, USA). To determine the experimental absorbance values, the average absorbance values of the triplicate samples and controls were calculated and subtracted from the absorbance values of the background control. The percentage of cytotoxicity was determined over the value of the high control (fixed to 100%).

### 3.19. Cell Proliferation

Cell proliferation was assessed after 3, 5 and 7 days of MSC culture using a Hoechst assay, which allows cell DNA quantification as previously described. Briefly, MSCs were harvested from 12-well plates and suspended in 100 µL of Hoechst buffer (10 mM TRIS, 1mM EDTA, and 0.1 M of NaCl, pH 7.4) before 5 series of freezing (liquid nitrogen)/thawing (60 °C, 5 min) cycles for lysing cells and releasing their DNA into solution. Black flat-bottom plates with low fluorescent background were used to perform the assay and a calf thymus DNA standard curve was used for the quantification. The samples were mixed with 2 µL of Hoechst solution (0.1 µg/mL in final concentration) and the measurements of DNA samples and standards were performed by fluorescence spectrophotometry (360 nm excitation/460 nm emissions, Varioskan^®^ Flash, Thermo). The DNA concentration (µg/mL) of each sample was based on its fluorescence measurement relative to the standard curve.

### 3.20. Cell Metabolic Activity

Cell metabolic activity was measured using an MTT [3-(4,5-dimethylthiazol-2-yl)-2,5-diphenyltetrazolium bromide] assay as described elsewhere. Fifty µL of MTT solution was added to 200 µL of cell culture medium. Briefly, MSCs were incubated for 4 h (5% CO_2_, 95% humidity at 37 °C) to allow the yellow dye to be transformed into blue formazan crystals by the mitochondrial dehydrogenases. The supernatant was removed and this insoluble product was protected from light and dissolved by the addition of 200 µL DMSO and gently mixed at 37 °C for 5 min. The supernatants were removed, protected from light, centrifuged, and their absorbance was read within 30 min using a Varioskan^®^ Flash (Thermo) at 540 nm. The control condition for MSC metabolic activity was used as the reference value.

### 3.21. Statistical Analysis

Results are expressed as the mean ± SD. Statistical analyses were performed with GraphPad Prism 6 (GraphPad Software, San Diego, CA, USA) using one-way ANOVA multiple comparisons followed by Tukey correction. *p* values were indicated in the legends if considered significant (* *p* < 0.01, ** *p* < 0.005).

## 4. Conclusions

This study revealed that the four Mediterranean sea cucumber species (*Holothuria poli*, *Holothuria tubulosa*, *Holothuria arguinensis,* and *Holothuria sanctori*) collected from three sampling sites on the Algerian west coast, have a high nutritional and pharmacological values and could be new attractive sources of EPA (5.07–8.76%), DHA (4.86–7.25%), phospholipids (55.20–69.85%), and ACE inhibitory peptides (IC_50_) (0.30 to 0.51 mg/mL). As the omega-3 and omega-6 fatty acids of sea cucumbers are obtained in a significant amount and susceptible to oxidation, proper handling of the sea cucumbers should be done to keep them fresh until consumed. Other dosage forms may be prepared such as tablets, capsules or emulsions containing sea cucumber extract which is rich in PUFAs (50.90–71.80%), to get the health benefits of the fatty acids in ameliorating the risk of certain diseases. This product may be consumed as a food supplement or nutraceutical with or without the addition of herbs to enhance the impact on human health. Their enzymatic hydrolysates obtained by Alcalase 2.4 L could be exploited as a new source of ACE inhibitory peptides and be incorporated as functional ingredients in nutraceuticals and pharmaceuticals due to their effectiveness in both the prevention and treatment of hypertension (regulating normal blood pressure in hypertensive humans). Also, they have a low-cost production, and a good tolerance by the human body. Our results on cytocompatibility assays showed that except for extreme concentrations (0.5 and 1 mg/mL), HTS was safe for human MSC viability and integrity. Indeed, in our experimental conditions, we demonstrated that nanoliposomes do not induce cytotoxicity and cell death of human MSCs and no perturbation of proliferation for all the times and the tested concentrations, as well as the combined nanoliposomes and HTS structures.

Those in vitro experiments strongly suggest that sea cucumbers lecithin nanoliposomes with or without HTS could be used as a natural carrier to deliver active substances into human MSCs cells. Therefore, studies are needed to investigate the peptide compositions in later work to reveal the most active peptides and further studies are needed with clinical trials for these marine-derived antihypertensive peptides.

## Figures and Tables

**Table 1:** Proximate composition (%) of the studied sea cucumbers body wall compared to other species from the Red Sea and Pacific Ocean. Mean ± SD [*n* = 3].

Species	Moisture (%)	Ash (%)	Protein (%)	Lipid (%)	References
*Holothuria poli*	67.76 ± 0.94	41.78 ± 1.82	69.34 ± 4.13	5.53 ± 0.59	This study
*Holothuria tubulosa*	61.85 ± 2.62	40.77 ± 0.60	49.26 ± 0.76	3.81 ± 0.25	This study
*Holothuria arguinensis*	64.55 ± 0.42	47.31 ± 0.88	66.41 ± 0.90	2.57 ± 0.28	This study
*Holothuria sanctori*	66.21 ± 1.34	31.58 ± 0.10	59.36 ± 2.32	3.07 ± 0.50	This study
*Holothuria arenicola*	72.12 ± 0.25	45.16 ± 0.22	44.56 ± 0.04	0.88 ± 0.05	[52]
*Actinopyga mauritiana*	76.54 ± 0.09	31.81 ± 0.34	66.86 ± 0.06	0.76 ± 0.02	[52]
*Holothuria leucospilota*	81.41 ± 0.60	4.3 ± 0.20	45.71 ± 0.20	4.60 ± 0.30	[51]
*Holothuria fuscogilva*	84.34 ± 0.72	30.45 ± 6.79	63.64 ± 4.56	1.12 ± 0.28	[34]
*Holothuria scabra*	85.76 ± 0.30	2.26 ± 0.15	43.43 ± 0.20	5.66 ± 0.09	[51]
*Thelonata ananas*	90.81 ± 2.08	37.40 ± 4.60	48.26 ± 2.32	2.35	[34]

**Table 2:** Lipid classes of the body wall of the studied sea cucumbers (%) by Iatroscan. Mean ± SD [*n* = 3].

Species	Neutral Lipids (%)	Phospholipids (%)	Glycolipids (%)
***H. poli***	4.56 ± 0.25	69.02 ± 0.78	23.54 ± 0.56
***H. tubulosa***	5.12 ± 0.18	79.23 ± 0.11	12.89 ± 0.69
***H. arguinensis***	6.43 ± 0.33	71.37 ± 0.58	19.79 ± 0.52
***H. sanctori***	3.55 ± 0.21	80.22 ± 0.32	13.63 ± 0.01

**Table 3:** Fatty acid compositions of the studied holothurians (%) analysed by CPG. Mean ± SD [*n* = 3]; ***n*.d.** not detected.

Fatty Acids	*H. poli*	*H. tubulosa*	*H. arguinensis*	*H. sanctori*
**C10:0**	1.43 ± 0.03	1.03 ± 0.07	1.32 ± 0.15	0.85 ± 0.00
**C12:0**	1.31 ± 0.03	0.76 ± 0.05	0.64 ± 0.15	0.93 ± 0.02
**C14:0**	0.66 ± 0.04	0.45 ± 0.05	1.87 ± 0.01	0.84 ± 0.03
**Iso-C16:0**	0.39 ± 0.00	***n*.d.**	0.45 ± 0.00	0.30 ± 0.01
**C16:0**	4.62 ± 0.19	2.68 ± 0.16	14.6 ± 0.00	4.15 ± 0.07
**C17:0**	1.24 ± 0.10	0.99 ± 0.07	0.95 ± 0.06	0.99 ± 0.02
**C18:0**	7.56 ± 0.51	5.87 ± 0.14	11.9 ± 0.07	7.92 ± 0.16
**C20:0**	0.87 ± 0.05	0.80 ± 0.05	0.81 ± 0.01	1.04 ± 0.02
**∑SFA**	18.10 ± 0.8	12.21 ± 0.36	32.38 ± 0.29	16.00 ± 0.17
**C14:1**	0.60 ± 0.02	0.39 ± 0.03	0.52 ± 0.00	0.40 ± 0.01
**C18:1*n*-9t**	3.21 ± 0.76	1.74 ± 0.09	4.82 ± 0.01	3.38 ± 0.67
**C18:1 *n*-9c**	6.12 ± 0.10	5.81 ± 0.14	6.58 ± 0.06	5.51 ± 0.12
**C20:1*n*-9**	3.40 ± 0.10	3.58 ± 0.22	2.38 ± 0.01	4.07 ± 0.09
**C20:1*n*-7**	2.26 ± 0.65	2.47 ± 0.06	2.02 ± 0.01	1.87 ± 0.23
**∑MUFA**	15.60 ± 0.65	14.39 ± 0.39	16.52 ± 0.99	14.48 ± 0.21
**C16:2*n*-6**	0.66 ± 0.03	0.51 ± 0.03	0.59 ± 0.00	0.63 ± 0.00
**C16:2*n*-4**	12.6 ± 0.44	15.0 ± 0.01	9.62 ± 0.03	11.2 ± 0.28
**C18:2*n*-6**	1.94 ± 0.04	2.11 ± 0.07	3.96 ± 0.02	2.61 ± 0.06
**C18:3*n*-6**	2.83 ± 0.07	2.95 ± 0.15	2.79 ± 0.04	4.10 ± 0.15
**C18:3*n*-3 (ALA)**	10.0 ± 0.35	11.0 ± 0.27	7.26 ± 0.03	9.33 ± 0.04
**C18:4*n*-3**	1.57 ± 0.04	1.55 ± 0.10	0.99 ± 0.01	1.44 ± 0.06
**C20:4*n*-6 (ARA)**	16.5 ± 0.58	18.9 ± 0.21	10.5 ± 0.03	15.3 ± 0.63
**C20:3*n*-3**	2.34 ± 0.03	2.11 ± 0.10	1.56 ± 0.02	3.24 ± 0.04
**C20:4*n*-3**	2.02 ± 0.09	1.87 ± 0.12	1.24 ± 0.01	1.35 ± 0.30
**C20:5*n*-3 (EPA)**	7.90 ± 0.28	8.76 ± 0.23	5.07 ± 0.04	8.62 ± 0.03
**C21:5*n*-3**	*n*.d.	*n*.d.	1.71 ± 0.02	3.15 ± 0.22
**C22:*4n*-6**	*n*.d.	0.36 ± 0.01	0.21 ± 0.00	*n*.d.
**C22:4*n*-3**	1.01 ± 0.04	1.02 ± 0.10	0.64 ± 0.01	1.60 ± 0.05
**C22:6*n*-3 (DHA)**	6.56 ± 0.53	7.25 ± 0.12	4.86 ± 0.12	4.97 ± 0.29
**∑PUFA**	60.78 ± 0.02	71.80 ± 0.69	50.90 ± 0.84	67.6 ± 0.23
**∑*n*-6**	21.3 ± 0.72	30.00 ± 0.35	17.40 ± 0.31	20.1 ± 0.53
**∑*n*-3**	31.5 ± 0.76	24.18 ± 0.30	23.30 ± 0.36	19.4 ± 0.43
**∑*n*-6/*n*-3**	0.67 ± 0.00	0.80 ± 0.00	0.74 ± 0.00	1.03 ± 0.07

**Table 4:** Melting temperatures and fusion enthalpies (∆H) of the studied sea cucumbers oils determined by DSC (−80–80 °C).

Species	Melting Temperatures (°C)	∆H (J/g)
***H. poli***	−41.71	3.74
***H. tubulosa***	2.41	0.87
***H. arguinensis***	−33.33	5.79
***H. sanctori***	−2.56	0.81

**Table 5:** General peak Wavenumber (cm^−1^) and assignments of the FTIR spectra of vibration of the four sea cucumbers oils in the wave number range of 4000–400 cm^−1^.

Assignment	Peak Wavenumber (cm^−1^)			
	*H. poli*	*H. tubulosa*	*H. arguinensis*	*H. sanctori*
υ_as_(CH_2_)	2925	2924	2922	2920
υ_sym_(CH*2*)	2854	2848	2848	2850
υ(C=O)	1739	1633	1629	1624
δ_as_(CH_2_)_sciss_	1471	1460	1442	1440
*p*(=CH) (*cis*)	1408	1404	1400	1402
γ(CH_2_)	1163	1163	1163	1163
υ(C**–**O**–**C)	1004	1022	1020	1024
γ(**–**HC=CH**–**) (*cis*-)	900	904	906	904
*p*(CH_2_)	754	754	752	756

as = antisymmetric, sym = symmetric; sciss = scissoring; υ = stretching; δ = deformation vibration (bend); *p* = rocking vibration; γ = out of plane deformation.

**Table 6:** Phospholipids compositions of the studied sea cucumber after acetone purification. Mean ± SD [*n* = 3].

	*H. poli*	*H. tubulosa*	*H. arguinensis*	*H. sanctori*
Phospholipids, PL (%)	55.20 ± 0.22	61.02 ± 0.17	63.09 ± 0.11	69.85 ± 0.02
Cardiolipin, CL (%)	10.14 ± 0.05	5.13 ± 1.24	6.50 ± 0.41	11.25 ± 0.41
Phosphatidylglycerol, PG (%)	14.38 ± 0.25	10.02 ± 0.53	14.08 ± 0,08	9.10 ± 0.42
Phosphatidylcholine, PC (%)	55.11 ± 0.41	58.57 ± 1.11	58.56 ± 0.80	51.48 ± 0.19
Phosphatidylethanolamine, PE (%)	8.10 ± 0.05	6.72 ± 0.00	6.31 ± 0.45	7.51 ± 0.04
Phosphatidylserine, PS (%)	6.47 ± 0.18	0.91 ± 0.10	1.12 ± 0.11	1.31 ± 0.03

**Table 7:** IC_50_ values obtained from the studied sea cucumbers hydrolysates (Alcalase 5%, <1 kDa).

Species	IC_50_ (mg/mL)
*H. poli*	0.51 ± 0.02
*H. tubulosa*	0.31 ± 0.00
*H. arguinensis*	0.35 ± 0.00
*H. sanctori*	0.30 ± 0.00

Mean ± SD [*n* = 3].
